# Real-World Impact of Metformin on Outcomes in Patients with Deficient DNA Mismatch Repair and Microsatellite Instability (dMMR/MSI) Colorectal Cancer Treated with Immune Checkpoint Inhibitors

**DOI:** 10.3390/cancers17243944

**Published:** 2025-12-10

**Authors:** Garima Gupta, Negar Sadeghipour, Fuat Bicer, Andrew Elliott, Andrew Hinton, Emil Lou, Ari M. Vanderwalde, Ahmet Anil Ozluk, Moh’d M. Khushman, Midhun Malla, Darryl Outlaw, Syed Qasim Hussaini, Bassel F. El-Rayes, Mehmet Akce

**Affiliations:** 1 Division of Hematology and Oncology, Department of Medicine, O’Neal Comprehensive Cancer Center, The University of Alabama at Birmingham, Birmingham, AL 35233, USA; 2 Caris Life Sciences, Phoenix, AZ 85040, USA; nsadeghipour@carisls.com (N.S.);; 3 Department of Medicine, The Ohio State University Comprehensive Cancer Center, Columbus, OH 43210, USA; 4 Division of Hematology, Oncology and Transplantation, University of Minnesota Twin Cities, Minneapolis, MN 55455, USA; 5 Division on Oncology, Washington University in St. Louis/Siteman Cancer Center, St. Louis, MO 63110, USA

**Keywords:** colorectal cancer, immune checkpoint inhibitors, metformin, DNA mismatch repair, microsatellite instability

## Abstract

**Background/Objectives**: Colorectal cancer (CRC) is the second leading cause of cancer-related death in the US. The presence of deficient DNA mismatch repair and microsatellite instability (dMMR/MSI) in CRC is linked to responses to immune checkpoint inhibitors (ICIs). This study investigates the impact of metformin on the tumor microenvironment (TME) and clinical outcomes of patients with dMMR/MSI CRC treated with ICIs, aiming to better understand its potential role in enhancing ICI efficacy. **Methods**: Of 25,011 CRC patients in Caris database, 47 received both metformin and ICI therapy (Met-ICI group), and 475 patients received ICI therapy alone (ICI group). Samples underwent genomic or transcriptome sequencing at Caris Life Sciences. Immune cell fractions were estimated using quanTIseq. Univariate and multivariate survival analyses were conducted using the Cox proportional model. **Results**: The TME analysis of CRC patient samples revealed that TMB-High (≥10 mut/Mb) was more prevalent in the “ICI” group compared to the “Met-ICI” group (99.1% vs. 95.6%, *p* = 0.036). Mutation rates for most genes between the two groups were similar, but CIC gene mutations were more common in the “ICI” group than in the “Met-ICI” group (23.2% vs. 4.8%, *p* = 0.006). No significant differences were observed in the PD-L1 positivity rate or immune checkpoint gene expression (including IDO1, IFNG, TIM3, and CTLA4). M1 macrophages and neutrophils showed the highest infiltration among immune cells. However, the fractions of infiltrated immune cells were similar between the two cohorts. Univariate and multivariate analyses showed that there was no significant difference in patient survival between “ICI” and “Met-ICI” cohorts. **Conclusions**: In this retrospective analysis of real-world clinical outcomes, the concurrent use of metformin with ICIs in patients with dMMR/MSI CRC did not reveal an impact on clinical outcomes.

## Simple Summary

Colorectal cancer (CRC) patients with deficient DNA mismatch repair and microsatellite instability (dMMR/MSI) are responsive to immune checkpoint inhibitors (ICIs). However, up to 50% of patients experience resistance. The anti-diabetic drug metformin has demonstrated anti-cancer properties in CRC. This study investigates the impact of metformin on the tumor microenvironment (TME) and clinical outcomes of dMMR CRC patients treated with ICIs. While we did not find any statistical differences in survival and TME characteristics for concomitant metformin and ICI use compared to ICI alone, we did make some key observations. We observed a positive association between the interferon-gamma score with survival in the overall cohort, and an association between PD-L1 positivity and survival in patients with dMMR/MSI CRC.

## 1. Introduction

Colorectal cancer (CRC) is the second leading cause of cancer-related death in the United States, with incidence rising among individuals under the age of 50 [1]. Molecular profiling of patients with stage IV disease is standard for guiding treatment. Up to 15% of CRC patients have microsatellite instability (MSI) due to deficient DNA mismatch repair (dMMR), a phenotype linked to high immune infiltration and checkpoint expression, contributing to improved responses with immune checkpoint inhibitors (ICIs) [2,3].

ICIs such as pembrolizumab, nivolumab ± ipilimumab, and dostarlimab have shown efficacy in dMMR/MSI CRC [4,5,6,7]. However, up to 50% of patients experience primary or secondary resistance. Identifying factors driving this variability is key to improving outcomes [8,9]. Tumor-infiltrating lymphocytes (TILs), cytotoxic T cells, and memory T cells in the tumor microenvironment (TME) are predictive of improved outcomes in CRC [10,11,12,13]. The NCCTG N0147 trial reported an association between lower TIL density and poorer survival in dMMR/MSI stage III CRC patients who received adjuvant chemotherapy [14].

The anti-diabetic drug metformin has demonstrated anti-cancer properties [15] including reduced incidence of colorectal adenomas and CRC [16,17,18] and longer overall survival (OS) in CRC patients with diabetes mellitus (DM) treated using this medication [19,20,21]. Preclinical studies have reported observations that metformin modulates the TME, in part by decreasing PD-L1 expression [22,23,24], enhancing oxygen consumption by CD8+ TILs [25,26], and by promoting T cell function [27,28]. However, clinical data on metformin combined with ICIs remain conflicting [29,30,31,32,33]. In this retrospective study, we report the impact of metformin on the TME and OS in dMMR/MSI CRC patients treated with ICIs.

## 2. Materials and Methods

### 2.1. Study Cohort

Patients whose tumor samples underwent genomic and transcriptomic molecular profiling at Caris Life Sciences (Phoenix, AZ, USA) were matched with insurance claims data, which detailed all recorded treatments over time. Inclusion criteria for metformin-treated patients required that the patient had received metformin within two months prior to the first dose of ICI therapy. This study was conducted in accordance with the guidelines of the Declaration of Helsinki, the Belmont Report, and US Common Rule. In compliance with policy 45 CFR 46.101 (b), this study was performed using retrospective and de-identified clinical data, and patient consent was not required.

### 2.2. Next-Generation Sequencing (NGS)

Depending on the time of testing, DNA sequencing was performed either using a 592-whole-gene panel on a NextSeq 500 System (RRID:SCR_014983), or using whole-exome sequencing (WES) on a NovaSeq 6000 System (RRID:SCR_016387) (Illumina, Inc., San Diego, CA). Genetic variants identified were interpreted by board-certified molecular geneticists. Although variants are categorized as ‘pathogenic,’ ‘likely pathogenic,’ ‘variant of unknown significance,’ ‘likely benign,’ or ‘benign,’ according to American College of Medical Genetics and Genomics standards, only pathogenic and likely pathogenic variants were included in comparative analyses.

Whole-transcriptome sequencing (WTS) was performed on a Novaseq 6000 System. Raw WTS data was demultiplexed by Illumina Dragen BioIT accelerator, trimmed, counted, and aligned to a human reference genome (hg19) by STAR aligner (RRID:SCR_004463) [34]. For transcription counting, transcripts per million (TPM) molecules were generated using the Salmon expression pipeline (RRID:SCR_017036) [35]. Relative abundance of immune cell infiltrates was calculated using quanTIseq as previously described [36]. The interferon-gamma score (IFN-γ) is a signature based on expression of 18 genes, which was calculated as previously described [37]. MPAS (MAPK Pathway Activity Score) calculation, which represents MAPK activation, was performed as previously described [38].

### 2.3. Tumor Mutational Burden (TMB)

TMB was measured by counting mutations found per tumor in the coding regions of genes analyzed (1.4 Mb for 592, 1.5 Mb for WES, and 25 Mb for Hybrid). For the 592-gene panel NGS assay, missense mutations were counted, and for WES/Hybrid, missense, nonsense, in-frame INDEL, and frameshift variants were counted. Filtering was performed to remove low-quality and low-depth variants or variants determined to be unreliable or unassociated with TMB. Presumed germline variants found in databases such as dbSNP “https://www.ncbi.nlm.nih.gov/snp/ (accessed on 4 December 2025)” (RRID:SCR_002338) and Genome Aggregation Database (gnomAD) ) “https://gnomad.broadinstitute.org/ (accessed on 4 December 2025)” (RRID:SCR_014964) and found in at least 10% of training samples were also filtered. A cutoff point of ≥10 mutations per MB was used based on the KEYNOTE-158 pembrolizumab trial [39].

### 2.4. Deficient Mismatch Repair/Microsatellite Instability (dMMR/MSI)

Multiple test platforms were used to determine the MSI or MMR status of profiled tumors. The three platforms generate highly concordant results [40], and in rare cases of discordant results, the MSI or MMR status of the tumor is determined in the order of descending priority: IHC, PCR, and NGS. Mismatch repair-deficient (dMMR) status was assigned to IHC test results if complete absence of any of four tested proteins (MLH1, MSH2, MSH6, or PMS2) was observed. MSI status was examined with NGS by using >7317 target microsatellite loci, and the cutoff for MSI designation was status > 45 altered loci, while tumors with <43 altered loci were assigned microsatellite stable (MSS) status. Multiplex PCR amplification of five mononucleotide repeat markers (BAT-25, BAT26, NR-21, NR24, and MONO-27) was performed using the MSI Analysis System (Promega, Madison, WI, USA), and MSI status was assigned to samples if two or more mononucleotide repeats were abnormal.

### 2.5. Immunohistochemistry (IHC)

IHC was performed on full formalin-fixed paraffin-embedded (FFPE) sections using automated staining techniques (Dako Link 48 Autostainer (Agilent, Santa Clara, CA, USA) or Ventana BenchMark Autostainers (Roche Diagnostics, Indianapolis, IN, USA)) and validated to CLIA/CAP and ISO standards. Results were classified as positive or negative based on marker-specific thresholds from clinical literature. A board-certified pathologist independently reviewed all results. PD-L1 was detected using the SP142 antibody, with positivity defined as ≥2+ membrane staining intensity and >1% of tumor cells stained.

### 2.6. Statistical Analysis

The prevalence of molecular alterations among cohorts was analyzed using Chi-square or Fisher’s Exact tests. Expression distribution among cohorts was analyzed using non-parametric Mann-Whitney U test. A *p*-value of <0.05 was considered a trending difference; *p* values were further corrected for multiple comparisons using the Benjamini–Hochberg method where applicable. *p* value of <0.05 was considered a significant difference.

### 2.7. Survival Analysis

Real-world OS information was obtained from insurance claims data for CRC patients with tumors previously analyzed by NGS. Survival durations were calculated from time from the first ICI administration to last contact. Kaplan–Meier (KM) estimates were calculated for cohorts defined by treatments or molecular characteristics. Hazard ratios (HRs) were calculated using the Cox proportional hazards model for both univariate and multivariate analyses. Significance was determined as *p* values < 0.05 (log-rank test).

## 3. Results

### 3.1. Cohort Characteristics

**Figure 1:**
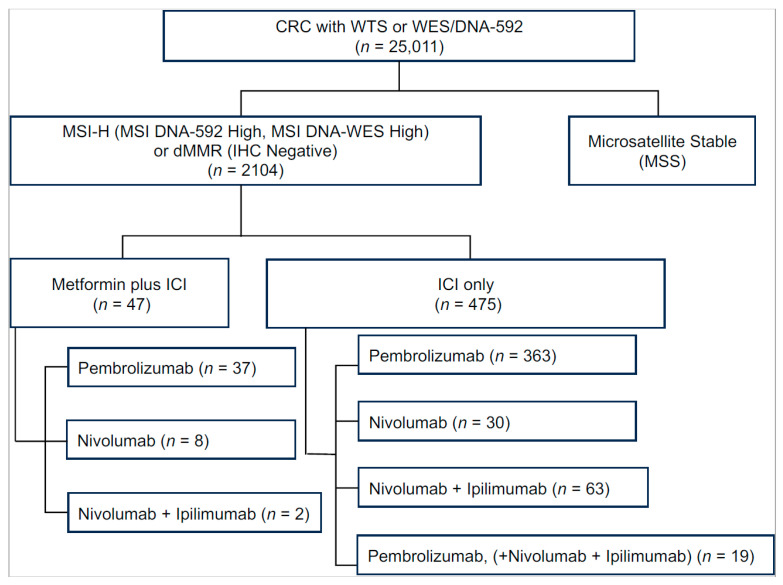
Number of CRC patients identified in Caris database. In the Metformin+ICI group, patients who received multiple immune checkpoint inhibitors were categorized according to the drug that had the longest overlap with metformin. Abbreviations: CRC, colorectal cancer; dMMR, deficient DNA mismatch repair; ICI, immune checkpoint inhibitor; MSI, microsatellite instability; WES, whole-exome sequencing; WTS, whole-transcriptome sequencing.

We retrospectively reviewed comprehensive molecular profiles from 25,011 CRC tumors (Figure 1). Among these, 2104 tumors (8.4%) were dMMR/MSI. Treatment records showed that 47 patients received both metformin and one or more ICI (pembrolizumab, nivolumab, or ipilimumab). This group was designated as “Met-ICI.” Patients were on metformin for an average ± STD of 5.14 ± 3.5 years for the “Met-ICI” group. In contrast, 475 metformin-naïve patients who received one or more of those ICIs were categorized as the “ICI” group.

In both the Met-ICI and ICI groups, pembrolizumab was the most commonly used ICI (78.7% vs. 76.4%), followed by nivolumab (17% vs. 6.3%), and a combination of nivolumab and ipilimumab (13.2% vs. 4%). In the ICI group, 4% received a combination of nivolumab and ipilimumab, along with pembrolizumab at some point during their treatment (Figure 1). Due to small numbers, patients who received dostarlimab, atezolizumab, or durvalumab (eight, six, and two, respectively) were excluded from further analysis.

The demographic characteristics are shown in Table 1. Compared to the “Met-ICI” group, the “ICI” group had a higher proportion of young adults (<40 years; 9.5% vs. 0%, *p* = 0.027) and elderly patients (>80 years; 21.3% vs. 6.4%, *p* = 0.014). In contrast, the 60–69 age range was more prevalent in the “Met-ICI” group (31.9% vs. 19.4%, *p* = 0.042). Approximately 80% of patients in both groups were white (*p* = 0.6), and 90% were non-Hispanic (*p* = 0.684). The distribution of primary tumor sites was similar between the groups, with about 50% of tumors being right-sided, 25% left-sided or transverse, and the remaining ones categorized as “not otherwise specified” or “others”. There was no significant difference in the distribution of specimen sites between the two groups, with approximately 60–70% of cases sampled from a local site (*p* = 0.372).

### 3.2. Molecular Landscape in DMMR/MSI CRC Patients Treated with Met-ICI and ICI

The rate of MSI was not significantly different between the “Met-ICI” group (87.2%, 39/47) and the ICI group (85.6%, 407/475) (*p* = 0.616). The IHC results for MMR proteins showed no significant difference in IHC-negative rates between the “Met-ICI” and “ICI” groups for any of the assessed proteins. MLH1 and PMS2 exhibited the highest rate of deficiency (70–88%), while MSH2 and MSH6 were positive in 79% to 95% of patients (Table S1).

**Figure 2:**
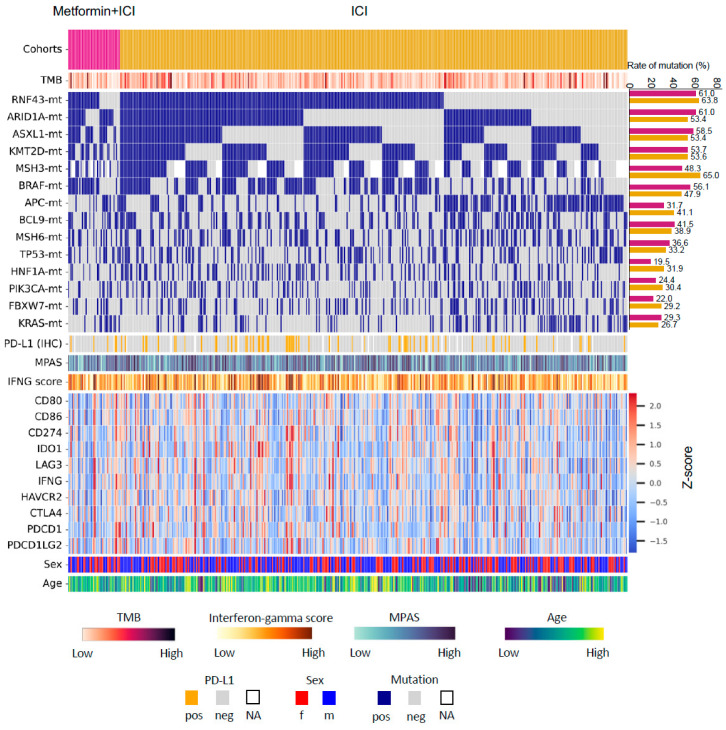
Oncoprint of colorectal cancer patients in this study. Only patients with available data from both next-generation sequencing and whole-transcriptome sequencing are presented. Abbreviations: Met, metformin; TMB, tumor mutational burden; ICI, immune checkpoint inhibitor; MPAS, MAPK pathway activity score; IFNG, Interferon-gamma; pos, positive; neg, negative; f, female; m, male; NA, not available.

Most genes showed similar mutation rates between the two groups (Figure 2, Table S2). The most frequent mutations in “Met-ICI” vs. “ICI” were RNF43 (61% and 63.8%), ARID1A (61% and 53.4%), ASXL1 (58.5% and 53.4%), KMT2D (53.7% and 53.6%), MSH3 (48.3% and 65%), and BRAF (56.1% and 47.9%) (each *p* > 0.05). CIC mutations exhibited a significantly higher prevalence in the “ICI” group compared to the “Met-ICI” group (23.2% vs. 4.8%, *p* = 0.006), while CHEK2 (4% vs. 12.2%, *p* = 0.023) and FOXA1 (1.2% vs. 7.3%, *p* = 0.005) showed the opposite trend. A transcriptional signature of MAPK kinase pathway activity, MPAS, was similarly distributed between two groups with no significant difference observed (Figure 2).

**Figure 3:**
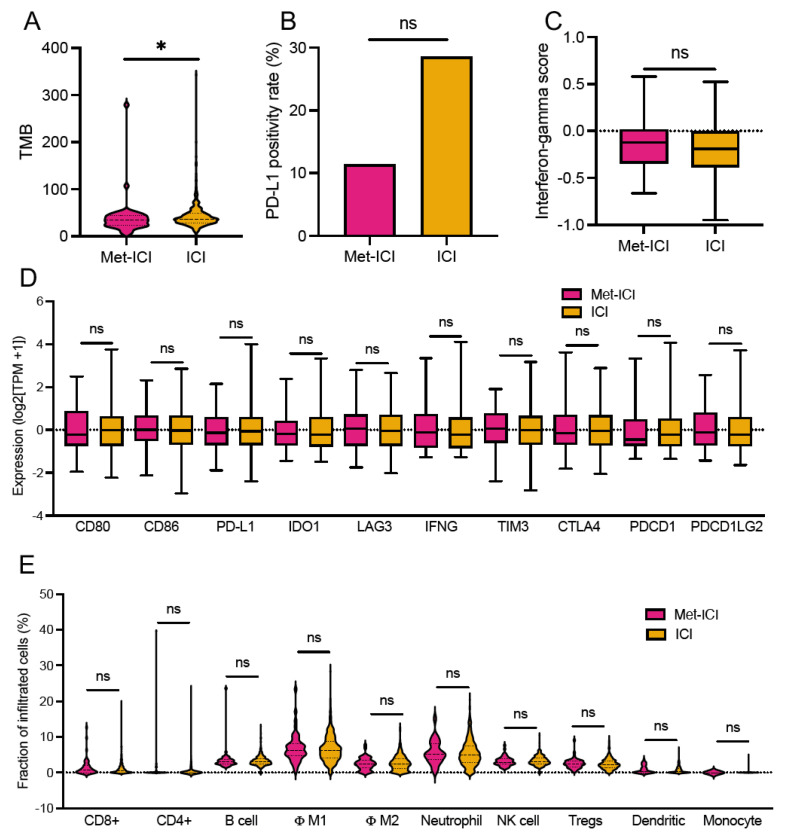
Tumor microenvironment of CRC patients. (**A**) Tumor mutational burden (TMB). (**B**) PD-L1. (**C**) Interferon-gamma score. (**D**) Expression of immune-related genes (transcripts per million, TPM). (**E**) Fraction of immune cells calculated by quanTIseq. Asterisks “*” indicate *p*-values < 0.05. Abbreviations: Met, metformin; TMB, tumor mutational burden; ICI, immune checkpoint inhibitor; MPAS, MAPK pathway activity score; Φ, macrophages; ns, not significant.

TMB-High was more prevalent in the “ICI” group (99.1%, 451/455) compared to the “Met-ICI” group (95.6%, 43/45) (*p* = 0.036). The mean ± STD (range) of TMB was 42.7 ± 26.6 (2–343 mut/Mb) in the “ICI” group, compared to 39.2 ± 40.1 (5–279 mut/Mb) in the “Met-ICI” group (*p* = 0.022) (Figure 3A). The prevalence of PD-L1 positivity measured by IHC was 11.4% (5/44) in the “Met-ICI” group compared to 28.5% (77/439) in the “ICI” group, with no significant difference between the two groups (*p* = 0.298) (Figure 3B). The IFN-γ score showed an average ± SD of -0.14 ± 0.27 in the “ICI” group and -0.18 ± 0.27 in the “Met-ICI” group, with no significant difference between the two groups (Figure 3C). None of the immune checkpoint genes exhibited significantly different expression profiles between the two cohorts (Figure 3D).

An analysis of immune cell infiltration using the quanTIseq deconvolution algorithm revealed that average infiltration levels were below 10% for many immune cell types (Figure 3E). In the “ICI” group, the highest infiltration was observed in M1 macrophages 6.8% ± 3.6% (median of 6.3%) and neutrophils 5.5% ± 3.5% (median of 4.9%). Other cell types had infiltration levels below 4%. Similarly, in the “Met-ICI” group, M1 macrophages had an average infiltration of 6.9% ± 4.1% (median of 4.2%), and neutrophils had an average of 5.6% ± 3.3% (median of 5%), representing the highest percentages of infiltrated cells. There was no significant difference between the infiltrated immune cell amounts in two groups.

### 3.3. Survival and Multivariate Analysis

**Figure 4:**
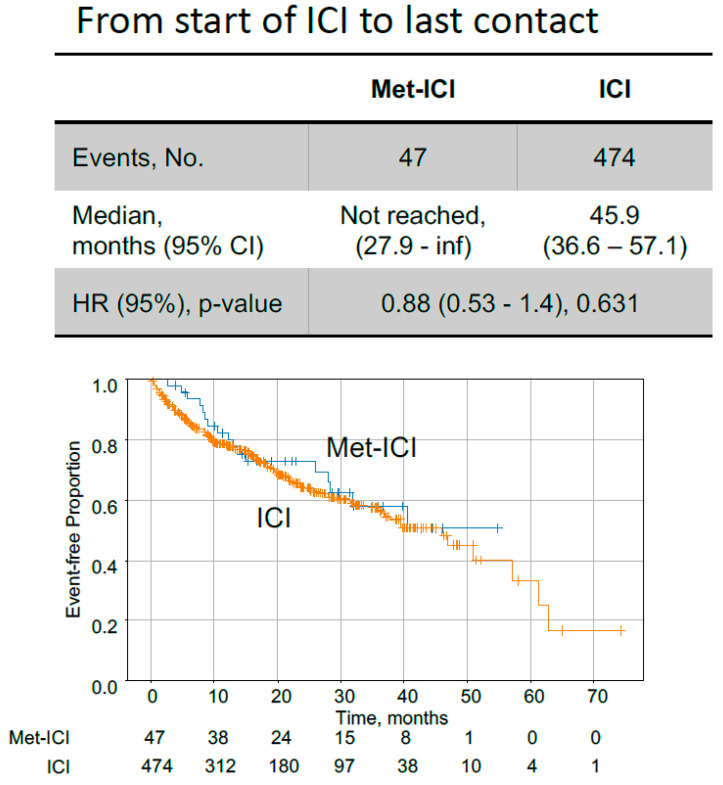
Kaplan–Meier curves comparing colorectal cancer patients with dMMR/MSI status, treated with metformin plus immune checkpoint inhibitors (Met-ICI) to patients treated only with immune checkpoint inhibitors (ICI). Time on ICI from start of treatment to last contact is shown. Abbreviations: met, metformin; ICI, immune checkpoint inhibitor; HR, hazard ratio; CI, confidence interval.

In CRC patients with dMMR/MSI status, the median time from the start of ICIs to last contact for the “Met-ICI” group was not reached, in contrast to the “ICI” group, for which this was 45.9 months (Figure 4). These times were not significantly different between the two groups. Survival analysis from tissue collection is limited by variable timing relative to ICI initiation and is thus provided as a sensitivity analysis, also resulting in no significant difference between the two groups (Figure S1). When KM analysis was performed for all members of the CRC cohort who received ICIs, but for whom MMR/MSI status was not consistently available, a statistically significant difference in OS was observed (Figure S2). The median survival time from the start of ICIs to last contact for “Met-ICI” vs. “ICI” group was 35.5 months vs. 17.9 months (Figure S2B).

**Figure 5:**
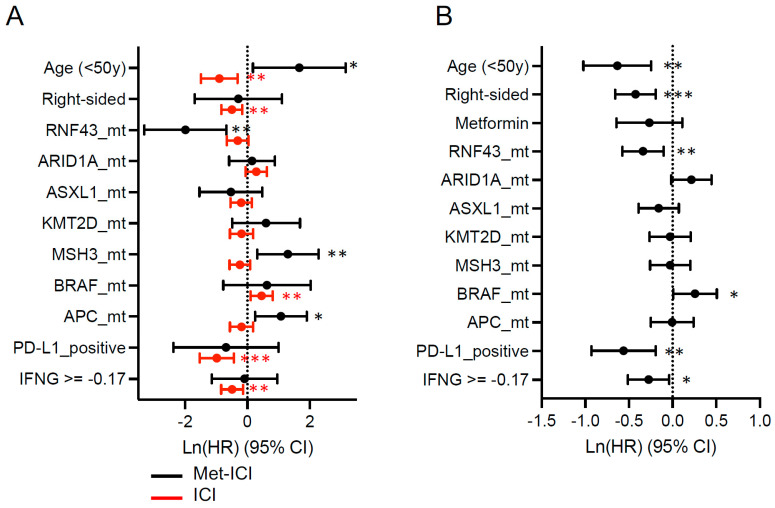
Forest plot showing the hazard ratio (HR) and 95% confidence interval (CI) for variables including common driver mutations (mt), PD-L1 positivity status, and interferon-gamma score with time from the start of pembrolizumab to last contact. Other covariates, such as age, sex and site were also tested. (**A**) Metformin plus ICI and ICI cohorts; (**B**) both cohorts together. Circles represent the hazard ratio, and the horizontal bars extend from the lower limit to the upper limit of the 95% confidence interval. For IFNG (interferon-gamma score), the median score of the two cohorts was used as the cutoff value. Asterisks represent the following *p*-values: * < 0.05, ** < 0.01, *** < 0.001.

The effect of individual biomarker alterations was analyzed in the “Met-ICI” and “ICI” groups separately (Figure 5A). In the “Met-ICI” group, age (< 50 years), MSH3 and APC mutations were independently associated with shorter OS and RNF43 mutations were independently associated with longer OS on ICI, while PD-L1 positivity and the IFN-γ score had no effect. In the “ICI” group, BRAF mutations were independently associated with shorter OS, while age (< 50 years), right-sided tumors, PD-L1 positivity and the IFN-γ score were associated with longer OS. In the multivariate analysis, we did not find that metformin affected survival; however, we noted the effect of other biomarkers in the overall cohort. BRAF mutations were independently associated with shorter OS, while mutations in RNF43 and ASXL1 and PD-L1 positivity and IFN-γ score were associated with longer OS (Figure 5B).

## 4. Discussion

This study examines whether metformin treatment in patients with dMMR/MSI CRC also treated with ICIs has a potential impact on genomic profiles, TME, or survival outcomes. The clinical and molecular characteristics of the patients in our study are in line with published findings, including the majority with a right-sided primary tumor and a high rate of BRAF, RNF43, ASLX1, and KMT2D mutations [8,9,41,42,43]. Increased expression (at least two-fold higher) of immune checkpoint genes including PD-L1 (CD274), CTLA4, LAG3, and IDO1 was noted in ICI-treated patients compared to CRC all-comers, consistent with published literature [8,9]. Interestingly, CD274 gene expression (Figure 3D) was similar between the two groups but PD-L1 positivity according to IHC was lower in patients in the “Met-ICI” group compared to those in the “ICI” group, *p* > 0.05 (Figure 3B). Consistent with prior preclinical studies showing that metformin downregulates PD-L1 expression across various cell lines [22,23,24], our finding in patient tumor samples warrants further validation in larger cohorts.

Given prior evidence showing that metformin positively affects CD8+ TIL survival and prevents their functional exhaustion [25,26,27,28], we expected to see higher CD8+ TIL infiltration in the “Met-ICI” group. While we did not observe this finding in our study, this should be further explored, as higher TIL density has previously been associated with higher responses and longer survival [14,44].

Metformin has been shown to improve survival in CRC patients with DM with variability among studies regarding its impact based on stage [19,20]. Particularly, it has been reported that metformin led to an increase in OS when used in combination with chemotherapy in patients with KRAS-mutated advanced CRC and DM [21]. However, there is conflicting outcome data with concomitant metformin and ICI use across various malignancies [29,30,31,32,33]. Although no significant difference was observed in our study between “Met-ICI” and “ICI” groups in dMMR/MSI patients (Figure 4), this observation may be constrained by the small cohort size. It is interesting to note that we observed a statistically significant difference in survival between “Met-ICI” vs. “ICI” in the overall ICI cohort of CRC patients treated with “Met-ICI” vs. “ICI” with undetermined MMR/MSI status (Figure S2). While this group was ~ threefold larger than the dMMR/MSI group, the lack of available MMR/MSI status for the entire ICI-treated cohort limits conclusions for particular subgroups based on key molecular determinants. While the use of ICIs in CRC is mainly limited to patients who are dMMR/MSI, ICIs are sometimes used as later-line therapy in MMR-proficient/MSS patients who have high PD-L1 and TMB scores. Thus, the observed effect in this homogenous cohort suggests that further investigations on larger cohorts are warranted to delineate possible mechanisms in which metformin might impact TME and ICI-related outcomes.

While the average IFN-γ score (−0.17) was higher in our dMMR/MSI population compared to that in the CRC all-comers (−0.39), we did not find a difference in IFN-γ between our two cohorts. We noted that the IFN-γ score was positively associated with survival in the “ICI” group as well as the overall cohort (Figure 5). BRAF mutations were also independently associated with shorter OS, consistent with existing literature regarding MSS CRC patients but not regarding dMMR/MSI patients [42,43]. We also noted that mutations in RNF43, as well as PD-L1 positivity, were associated with longer OS. While data regarding the predictive value of PD-L1 for response to ICIs across cancer types is variable [45], we did find an association between PD-L1 positivity and survival in our patients with dMMR/MSI CRC.

A primary limitation of our study is the small cohort size of patients who received metformin and ICI (9%), which increases the risk of a type II error and therefore limits conclusions about the lack of observed differences between these groups until a larger sample size may provide statistical power to support this observation. Additionally, this study analyzes retrospective data and assessment of survival is based on last contact with the health care system as per insurance claims data and may not be representative of real OS. Retrospective studies are also vulnerable to inherent biases from data collected without prior study design. As such, this study does not account for multiple variables which may impact the molecular landscape of the sequenced samples. There may also be other confounders not included in the multivariate Cox models due to the lack of some clinical information such as the diagnosis/status of DM, other concurrent diagnoses, treatment regimens, and serious adverse effects. Although metformin is typically only given to patients with confirmed DM, the claims database does not have complete information about disease state or other clinical conditions for the metformin-treated subcohort. Furthermore, different underlying prognoses associated with the DM disease state, or lack thereof in the control ICI group, could be significant confounding factors mediating ICI response. Although we did not have staging information available, most patients likely had advanced disease since ICIs are currently approved for metastatic dMMR/MSI CRC and comprehensive molecular profiling is more commonly utilized in the advanced setting. Furthermore, we did not have MLH1 promoter hypermethylation status or germline status available; therefore, we were unable to evaluate any differences associated with metformin use between germline and sporadic patients. Thus, the inherent tissue heterogeneity from this exploratory study limits conclusive observations, and future studies to address some questions raised herein would benefit from larger cohorts parsed by histological and molecular subtypes, and other specific variables. Conclusions that can be made about prognostic or predictive value are limited to the generation of new hypotheses and suggestions for directions of future studies or clinical trials.

## 5. Conclusions

There were no observed statistical differences in molecular features or survival with concomitant metformin and ICI use compared to ICI alone in patients with dMMR/MSI CRC. In the overall ICI-treated CRC cohort where MMR/MSI status was not consistently available, treatment with “Met-ICI” was associated with significantly longer survival compared to “ICI” alone, suggesting further investigation to explore potential roles of metformin in TME modulation and ICI response.

## Figures and Tables

**Table 1:** Clinicopathologic characteristics of treated colorectal cancer patients used in this study. Comparison of cohorts treated with metformin + immune checkpoint inhibitor (Met-ICI) and immune checkpoint inhibitor (ICI). Abbreviations: NOS, not otherwise specified. #, Self-reported race and ethnicity data were only available for about 80% of the patients.

		Met-ICI (*n* = 47) Cases (%)	ICI (*n* = 475) Cases (%)	Chi-Square *p*-Value
**Sex**	Male	24 (51.1)	193 (40.6)	0.166
**Age at collection (years)**	<40	0 (0)	45 (9.5)	0.027
	40–49	5 (10.6)	55 (11.6)	0.847
	50–59	8 (17)	63 (13.3)	0.473
	60–69	15 (31.9)	92 (19.4)	0.042
	70–79	16 (34)	119 (25.1)	0.179
	>80	3 (6.4)	101 (21.3)	0.014
**Race #**	White	34 (85)	326 (79.1)	0.600
	African American	4 (10)	51 (12.4)	0.635
	Asian	2 (5)	11 (2.7)	0.415
	Others	0 (0)	24 (5.8)	0.114
**Ethnicity #**	Non-Hispanic	36 (90)	353 (88)	0.684
	Hispanic	4 (10)	49 (12.2)	0.684
**Location of primary tumor**	Colon, left-sided	9 (19.1)	66 (13.9)	0.327
	Colon, right-sided	24 (51.1)	228 (48)	0.688
	Colon, transverse	3 (6.4)	55 (11.6)	0.279
	Colon, NOS	9 (19.1)	87 (18.3)	0.888
	Others	2 (4.3)	39 (8.2)	0.336
**Specimen site**	Local	32 (68.1)	292 (61.5)	0.372
	Distant	15 (31.9)	183 (38.5)	0.372

## Data Availability

The deidentified sequencing data are owned by Caris Life Sciences. The datasets generated during and analyzed during the current study are available from the authors upon reasonable request and with the permission of Caris Life Sciences. Qualified researchers may contact the corresponding author with their request.

## Supplementary Materials

The following supporting information can be downloaded at https://www.mdpi.com/article/10.3390/cancers17243944/s1, Table S1: Prevalence of mismatch repair proteins in cohorts treated with metformin + immune checkpoint inhibitor (Met-ICI) and immune checkpoint inhibitor (ICI), as detected by immunohistochemistry. Table S2: Mutation landscape in cohorts treated with metformin + immune checkpoint inhibitor (Met-ICI) and immune checkpoint inhibitor (ICI). Figure S1: Kaplan–Meier curves comparing patients treated with metformin plus immune checkpoint inhibitors (Met-ICI) to patients treated only with immune checkpoint inhibitors (ICI). Survival time shown from sample collection date to last contact. Figure S2: Kaplan–Meier curves comparing patients treated with metformin plus immune checkpoint inhibitors (Met-ICI) to patients treated only with immune checkpoint inhibitors (ICI) in the overall ICI cohort of CRC patients with undetermined MMR/MSI status (A) Survival time from sample collection date to last contact, and (B) time on ICI from start of treatment to last contact. Abbreviations: met, metformin; ICI, immune checkpoint inhibitor; HR, hazard ratio; CRC, colorectal cancer; MMR, mismatch repair; MSI, microsatellite instability.

## Abbreviations

The following abbreviations are used in this manuscript:

CRC	Colorectal cancer
ICIs	Immune checkpoint inhibitors
MSI	Microsatellite instability
dMMR	Deficient DNA mismatch repair
TME	Tumor microenvironment
OS	Overall survival
Met-ICI	Group of patients that received both metformin and one or more ICI
ICI	Group of metformin-naïve patients who received one or more ICI
