# Supplementary material for: Real-World Impact of Metformin on Outcomes in Patients with Deficient DNA Mismatch Repair and Microsatellite Instability (dMMR/MSI) Colorectal Cancer Treated with Immune Checkpoint Inhibitors
Source: Cancers (Basel). 2025 Dec 10;17(24):3944. doi: 10.3390/cancers17243944 (PMC12731130; doi:10.3390/cancers17243944)
Supplement: Supplementary file 1 [file cancers-17-03944-s001.zip › Supplemental figures.pdf]

From collection to last contact

	Met-ICI	ICI
Events, No.	47	475
Median, months (95% CI)	51.1, (33.1 - inf)	53.2, (44.3 – 69.1)
HR (95%), p-value	0.91 (0.55 – 1.5), 0.714	

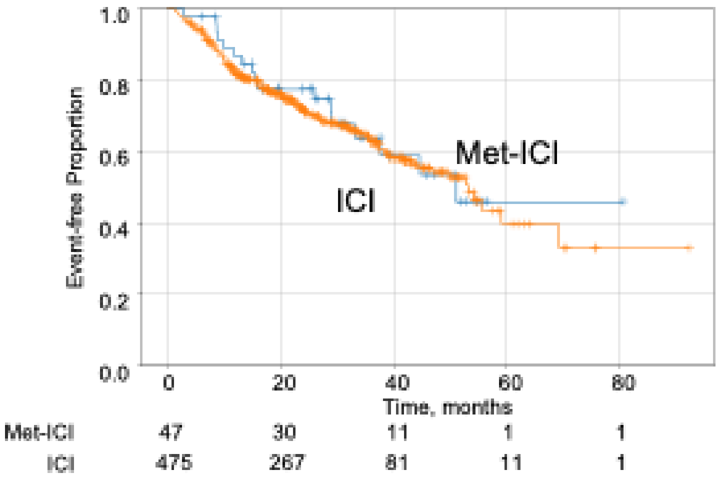

Figure S1. Kaplan-Meier curves showing patients treated with metformin plus immune checkpoint inhibitors (Met-ICI) compared to patients treated only with immune checkpoint inhibitors (ICI). Survival time shown from sample collection date to last contact. Abbreviations: met, metformin; ICI, immune checkpoint inhibitor; HR, hazard ratio.

A From collection to last contact

	Met-ICI	ICI
Events, No.	146	1495
Median, months (95% CI)	51.1, (42.4 – 55.7)	35.8, (33.8 – 38.0)
HR (95%), p-value	0.68 (0.53 – 0.87), 0.003	

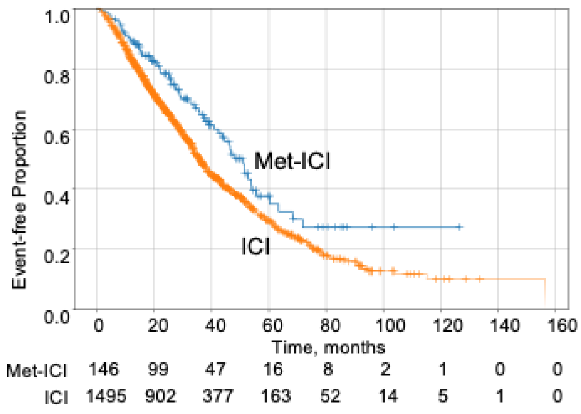

B From start of ICI to last contact

	Met-ICI	ICI
Events, No.	146	1486
Median, months (95% CI)	35.5, (26.5 – 50.5)	17.9, (15.5 – 20.6)
HR (95%), p-value	0.67 (0.48 – 0.79), < 0.001	

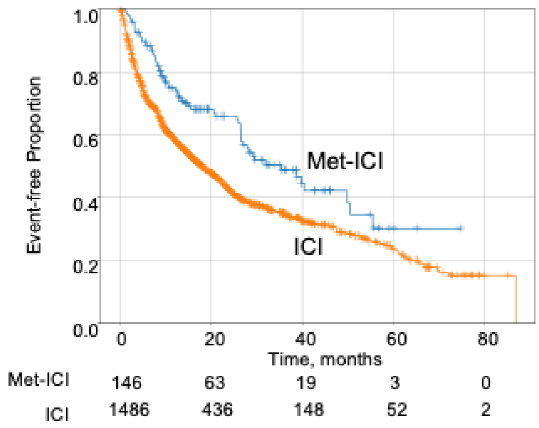

Figure S2. Kaplan-Meier curves comparing patients treated with metformin plus immune checkpoint inhibitors (Met-ICI) to patients treated only with immune checkpoint inhibitors (ICI) in the overall ICI cohort of CRC patients with undetermined MMR/MSI status (A) Survival time from sample collection date to last contact, and (B) time on ICI from start of treatment to last contact. Abbreviations: met, metformin; ICI, immune checkpoint inhibitor; HR, hazard ratio; CRC, colorectal cancer; MMR, mismatch repair; MSI, microsatellite instability.
