# Supplementary material for: Real-World Impact of Metformin on Outcomes in Patients with Deficient DNA Mismatch Repair and Microsatellite Instability (dMMR/MSI) Colorectal Cancer Treated with Immune Checkpoint Inhibitors
Source: Cancers (Basel). 2025 Dec 10;17(24):3944. doi: 10.3390/cancers17243944 (PMC12731130; doi:10.3390/cancers17243944)
Supplement: Supplementary file 1 [file cancers-17-03944-s001.zip › Tabe S1.pdf]

	Met-ICI			ICI			Chi-Square
**Feature**	Positive	Negative	% positive	Positive	Negative	% positive	P-value
MLH1	8	31	20.5	115	270	29.8	0.219
MSH2	39	2	95.1	329	60	84.6	0.067
PMS2	5	36	12.2	81	304	21.0	0.179
MSH6	36	5	87.8	305	81	79.0	0.182

Table S1. Prevalence of mismatch repair proteins in cohorts treated with metformin + immune checkpoint inhibitor (Met-ICI) and immune checkpoint inhibitor (ICI), as detected by immunohistochemistry.
