# Supplementary material for: Real-World Impact of Metformin on Outcomes in Patients with Deficient DNA Mismatch Repair and Microsatellite Instability (dMMR/MSI) Colorectal Cancer Treated with Immune Checkpoint Inhibitors
Source: Cancers (Basel). 2025 Dec 10;17(24):3944. doi: 10.3390/cancers17243944 (PMC12731130; doi:10.3390/cancers17243944)
Supplement: Supplementary file 1 [file cancers-17-03944-s001.zip › Table S2.pdf]

	Met-ICI			ICI			Chi-Square
Mutation	Mutant	Wild-type	% Mutant	Mutant	Wild-type	% Mutant	P-value
RNF43	25	16	61	256	145	63.8	0.716
ARID1A	25	16	61	214	187	53.4	0.351
ASXL1	24	17	58.5	214	187	53.4	0.527
KMT2D	22	19	53.7	215	186	53.6	0.995
MSH3	14	15	48.3	202	109	65	0.07
BRAF	23	18	56.1	192	209	47.9	0.316
APC	13	28	31.7	165	236	41.1	0.24
BCL9	17	24	41.5	156	245	38.9	0.748
TP53	15	26	36.6	133	268	33.2	0.658
HNF1A	8	33	19.5	128	273	31.9	0.101
PIK3CA	10	31	24.4	122	279	30.4	0.421
FBXW7	9	32	22	117	284	29.2	0.329
KRAS	12	29	29.3	107	294	26.7	0.722
CREBBP	10	31	24.4	108	293	26.9	0.726
SMAD4	2	39	4.9	42	359	10.5	0.254
BRCA1	4	37	9.8	27	374	6.7	0.47
BRCA2	9	32	22	101	300	25.2	0.648
PTEN	4	37	9.8	86	315	21.4	0.076
AKT1	1	40	2.4	14	387	3.5	0.723
MGA	9	20	31	58	253	18.6	0.109
CHEK2	5	36	12.2	12	289	4	**0.023**
FOXA1	3	38	7.3	5	396	1.2	**0.005**
CIC	2	39	4.8	93	308	23.2	**0.006**

Table S2. Mutation landscape in cohorts treated with metformin + immune checkpoint inhibitor (Met-ICI) and immune checkpoint inhibitor (ICI).
